# Evaluation of nonmotor symptoms in myasthenia gravis patients

**DOI:** 10.55730/1300-0144.5951

**Published:** 2024-10-19

**Authors:** Aysel TEKEŞİN, Çağla ŞİŞMAN, Ufuk Emre TOPRAK, Alena SAR, Burcu AKALIN, Goncagül KOŞARGELİR, Melih TÜTÜNCÜ, Enes YİĞİT

**Affiliations:** 1 Department of Neurology, İstanbul Training and Research Hospital, Health Sciences University, İstanbul, Turkiye; 2 Department of Otolaryngology-Head and Neck Surgery, İstanbul Training and Research Hospital, Health Sciences University, İstanbul, Turkiye; 3 Department of Neurology, Cerrahpaşa Faculty of Medicine, İstanbul University-Cerrahpaşa, İstanbul, Turkiye

**Keywords:** Myasthenia gravis, nonmotor symptoms, systemic impact

## Abstract

**Background/aim:**

Myasthenia gravis (MG) is chronic autoimmune disorder characterized primarily by muscle weakness and fatigue, due to autoantibodies targeting acetylcholine receptor at neuromuscular junction. Herein, it was aimed to evaluate nonmotor symptoms in MG patients and compare them with disease duration, severity, and clinical features.

**Materials and methods:**

This prospective study was conducted with two groups: 35 MG patients and 35 healthy volunteers. The patient group comprised 17 females and 18 males with a mean age of 52.9 ± 13.6 years. The control group comprised 23 females and 12 males with a mean age of 46.9 ± 12.2 years. The Myasthenia Gravis Foundation of America clinical classification and Myasthenia Gravis Composite and Turkish version of the Myasthenia Gravis Quality of Life 15 scales were applied to the patient group. To assess nonmotor functions, questionnaires evaluating the symptoms of headache, pain, depression, anxiety, sleep, physical activity, cognition, smell, and taste were applied to both the patient and control groups.

**Results:**

The results revealed prevalent and diverse nonmotor symptoms in the MG group. The Montreal Cognitive Assessment (MoCA) score and degree of olfactory function in cinnamon was statistically significantly (p < 0.05) lower in the patient group than in the control group. The degree of impairment on the MoCA, headache rate, Beck depression score, depression rate, Beck anxiety score, anxiety rate, Pittsburgh Sleep Quality Index (PSQI) score, and poor sleep quality rate were significantly higher (p < 0.05) in the patient group than in the control group.

**Conclusion:**

In conclusion, this study emphasizes the importance of recognizing and managing nonmotor symptoms in MG patients, advocating for a multidisciplinary approach to care that addresses both motor and nonmotor manifestations. By expanding our understanding of the systemic impact of MG and exploring new treatment modalities, we can improve the lives of those living with this complex autoimmune disorder. Further research is needed to elucidate the pathogenic mechanisms underlying these nonmotor manifestations, which will be pivotal in developing more targeted and effective therapeutic strategies.

## 1. Introduction

Myasthenia gravis (MG) is a chronic autoimmune disorder characterized primarily by muscle weakness and fatigue, attributed to the disruption of neuromuscular transmission due to autoantibodies targeting the acetylcholine receptor, muscle-specific kinase (MuSK), and lipoprotein receptor-related protein 4 (LRP-4) at the neuromuscular junction and causing structural and functional changes in the postsynaptic membrane [1–11]. While clinical focus has traditionally been on the motor symptoms of MG, which include fluctuating weakness of ocular, bulbar, and limb muscles throughout the day due to the impairment in neuromuscular transmission, emerging evidence has suggested that nonmotor symptoms also play a significant role in the burden of the disease [2,5,7,11].

It was stated that many different nonmotor symptoms, in addition to fatigue and skeletal muscle weakness, may also be observed during the course of the disease. Nonmotor findings can be seen as symptomatic findings of both the nervous system and other systems. These nonmotor symptoms in MG are diverse, ranging from autonomic dysfunctions, such as taste disorders, to systemic manifestations like alopecia areata, headache, memory disorders, fatigue, vitiligo, sleep, anxiety, and mood disturbances [12–26]. These symptoms often exacerbate the disease’s impact on patients’ quality of life, but remain underrecognized and poorly characterized in the clinical setting. Notably, the prevalence of accompanying autoimmune diseases, such as Graves’ disease, Hashimoto’s thyroiditis, rheumatoid arthritis (RA), and systemic lupus erythematosus (SLE), inflammatory myopathies, and myocarditis, underscores the systemic nature of MG and highlights the potential for broader immune dysregulation [17,22,25,27–34].

Despite the acknowledged presence of these nonmotor symptoms, detailed evaluations of MG are scarce in the literature. Studies that have explored these aspects provide preliminary insights, indicating significant prevalence rates of symptoms such as pain, headache, sensory impairments and autonomic dysfunction, sleep disturbance, and cognitive and psychosocial issues among MG patients [12,14]. For instance, research has shown that approximately 25% of patients with thymoma-associated MG present with one or more nonmotor symptoms, with alopecia areata and taste disorders being particularly common [13–16,19,20]. Furthermore, the correlation between olfactory function deterioration and MG severity suggests a broader spectrum of sensory involvement in MG than previously appreciated [35–38].

The pathogenesis of nonmotor symptoms in MG has not been fully elucidated but is thought to involve mechanisms both related and unrelated to the autoantibody-mediated attack on the neuromuscular junction. Antibody-related damage and CD8+ T cell-mediated cytotoxicity have been proposed as underlying mechanisms, affecting not only muscle function but also potentially contributing to the systemic and neurological manifestations observed in MG patients [13–15].

Given the significant gap in the literature regarding the comprehensive evaluation of nonmotor symptoms in MG, this study aimed to fill this void by systematically assessing these symptoms across both the systemic and neurological domains. By correlating these findings with disease duration, severity, and clinical features, it was sought to provide a more holistic understanding of the impact of MG on patients, thereby providing more targeted and effective management strategies.

## 2. Materials and methods

This study was conducted prospectively at the Neurology Outpatient Clinic of İstanbul Training and Research Hospital. Included were 35 patients diagnosed with MG among the 151 previously diagnosed MG patients admitted to the Neurology Outpatient Clinic between November 2023 and August 2024. A significant number of the patients were excluded, as they did not meet the inclusion criteria. Hence, 35 patients affected by MG, comprising 17 females and 18 males, were included as the patient group. The control group comprised 35 age- and sex-matched healthy volunteers, who were hospital employees or their relatives, of which 23 were female and 12 were male. Included in the study were patients between 18 and 70 years of age, who applied to the neurology outpatient clinic and were followed-up with the diagnosis of MG based on clinical, laboratory and electrophysiological data, who did not have a history of serious heart diseases, neurological diseases other than MG, psychiatric disorders, or chronic systemic diseases (cancer, chronic kidney failure, liver disease). Patients who were able to complete the required questionnaires and forms, had their clinical characteristics recorded, were not pregnant, and consented to participate were included in the study.

Exclusion criteria included having a history of any accompanying neurological diseases, such as known polyneuropathy, dementia, previous stroke, Parkinson’s disease; disease or drug use that may cause autonomic system dysfunction; known serious heart diseases; psychiatric disorders and chronic systemic diseases (cancer, chronic kidney failure, liver disease); and being under the age of 18 and over the age of 70. Patients who could not complete the questionnaires and forms required for the study, whose clinical characteristics could not be recorded, who were pregnant, and who did not agree to participate were excluded from the study.

Some general information like age, sex, previous clinical history, such as MG disease duration, MG onset age, MG onset type, MG severity, and electromyography and acetylcholine receptor antibody results available in the system were recorded for all the patients. The patients were questioned in detail regarding systemic and neurological symptoms. In the systemic questioning, the cardiovascular system, skin findings, presence of depression and anxiety disorders were evaluated. In the neurologic questioning, headache, dizziness, autonomic symptoms, sleep disorders, sensory complaints like odor and taste, cognitive functions, and other neurological symptoms were asked about.

The Myasthenia Gravis Foundation of America (MGFA) clinical classification, Myasthenia Gravis Composite (MGC), Turkish version of the Myasthenia Gravis Quality of Life 15 (MG-QOL15-T) [39] were applied to the patients to evaluate MG. For nonmotor functions, in both the patients and controls, the following questionnaires, in which the symptoms of the neurological system were evaluated separately (headache, pain, depression, anxiety, sleep, physical activity, basic activities of daily living, cognition, smell, taste, etc.) were used:

- The Beck Depression Inventory (BDI), which is a 21-item, self-report rating inventory that measures characteristic attitudes and symptoms of depression.
- The Beck Anxiety Inventory (BAI), which is a formative assessment and rating scale of anxiety.
- The Pittsburgh Sleep Quality Index (PSQI), which is a self-rated questionnaire that assesses sleep quality and disturbances over a one-month interval.
- The McGill–Melzack Pain Questionnaire for pain assessment.
- The Katz Index of Independence in Activities of Daily Living (Katz ADL), which assesses the basic activities of daily living.
- The International Physical Activity Questionnaire-Short Form, which assesses physical activity.
- The Montreal Cognitive Assessment (MoCA) test, which was validated as a highly sensitive tool for early detection of mild cognitive impairment and the early onset of dementia.
- Olfactory function assessment, which was carried out by means of the Connecticut Chemosensory Clinical Research Center (CCCRC) oronasal olfaction test [40]. The CCCRC test is a simple, validated, and widely used test. It includes odor discrimination and identification tests. Discrimination was performed with nine serial dilutions of butanol. The identification was made with the scents of cinnamon, baby powder, soap, peanut butter, Vicks, cocoa, coffee, and naphthalene.
- The Gustatory Function Assessment test, which was used to evaluate gustatory function. It is a standardized and validated test, which investigates the ability to perceive four basic tastes (sweet, salty, sour, and bitter) [41].

In the MG disease classification, the MGFA clinical classification was used, which is a classification that reveals the severity of the disease, enables meaningful comparison of patient subgroups, and evaluates prognosis and different responses to different therapies.

MGC scoring was used to evaluate the signs and symptoms, that is, the clinical status of the disease.

Additionally, the MG-QOL15-T was used to evaluate the patients’ quality of life. The scale consists of 15 items, and each item is scored between 0 and 4. The lowest score is 0, and the highest is 60. A high score indicates poor quality of life.

All the surveys, forms, and tests were conducted face to face by certain physicians in a quiet room. The clinical characteristics of the results obtained, the treatment responses, and their relationship with prognosis were compared.

Using all of these, it was aimed to evaluate the nonmotor symptoms of the systemic (cardiovascular system, skin findings, mood, etc.) and neurological (headache, sleep disorders, taste and smell functions) systems in MG patients and compare them with the disease duration, severity, and clinical features, and then compare them with the control group.

### 2.1. Statistical analysis

The mean, standard deviation (SD), median, lowest, highest, frequency, and ratio values were used in the descriptive statistics of the data. The distribution of the variables was measured using the Kolmogorov–Smirnov and Shapiro–Wilk tests. The Mann–Whitney U test was used in the analysis of the independent quantitative data with nonnormal distribution. The chi-squared test was used in the analysis of the independent qualitative data, and the Fischer’s test was used when the chi-squared test conditions were not met. p < 0.05 was accepted as statistically significant. IBM SPSS Statistics for Windows 28.0 (IBM Corp., Armonk, NY, USA) was used in the data analyses.

### 2.2. Ethical approval

The study was approved by the İstanbul Training and Research Hospital Ethics Committee under number 305, dated November 10, 2023. All the participants provided written informed consent prior to participation in the study.

## 3. Results

The clinical characteristics of the patients are shown in Table 1.

The patient group comprised 35 MG patients, of whom 17 (48.6%) were female and 18 (51.4%) were male, with a mean age of 52.9 ± 13.6 years. The control group consisted of 35 healthy individuals, of whom 23 (65.7%) were women and 12 (34.3%) were men, with a mean age of 46.9 ± 12.2 years (Table 2).

The age and sex distribution did not differ significantly between the control and patient groups (p > 0.05). The rate of comorbidities in the patient group was significantly higher than in the control group (p < 0.05) (Table 2).

The MoCA score in the patient group was significantly lower than in the control group (p < 0.05). The BDI and BAI scores in the patient group were significantly higher than in the control group (p < 0.05). The depression and anxiety rates in the patient group were significantly higher than in the control group (p < 0.05) (Table 2).

The PSQI score in the patient group was significantly higher than in the control group (p < 0.05). The rate of poor sleep quality in the patient group was significantly higher than in the control group. There was no significant difference in the olfactory perception between the control and patient groups (p > 0.05). Similarly, there was no significant difference in the odor concentration required to detect smells between the control and patient groups (p > 0.05) (Table 2).

The headache rate in the patient group was significantly higher than in the control group (p < 0.05). The degree of physical activity did not differ significantly between the control and patient groups (p > 0.05). Similarly, the McGill–Melzack pain rate did not differ significantly between the control and patient groups (p > 0.05) (Table 3).

The degree of olfactory function with baby powder, coffee, Vicks, cocoa, soap, naphthalene, peanut butter; and the degree of taste function for the four basic tastes, bitter, sour, sweet and salty, were not significantly different between the control and patient groups (p > 0.05). Interestingly, the degree of olfactory function with cinnamon in the patient group was significantly lower than in the control group (p < 0.05). The rate of allergic rhinitis during examination did not differ significantly between the control and patient groups (p > 0.05) (Table 3).

The MG composite (MGC) scores did not differ significantly between the groups with and without headache; with and without McGill–Melzack pain; with a normal and impaired MoCA degree; with inactive physical activity and minimal physical activity; with and without anxiety; with normal and poor PSQI scores; with and without normal olfactory function; with odor detection at concentrations ≤5 and >6; with ocular and generalized MG; with negative and positive antibodies; with and without thymectomy, with and without intravenous immunoglobulin (IVIG) treatment; and with and without intensive care unit (ICU) admission (p > 0.05) (Table 4).

The MGC score in the depression group was significantly higher than in the nondepression group (p < 0.05). The MGC score in the group with allergic rhinitis on examination was significantly lower than in the group without allergic rhinitis on examination (p < 0.05) (Table 4).

The MGFA scores did not differ significantly between the groups with and without headache; with and without McGill–Melzack pain; with a normal and impaired MoCA degree; with inactive and minimally active physical activity; with and without depression; with and without anxiety; with normal and poor PSQI scores; with and without normal olfactory function; with odor detection at concentrations of ≤ 5 and >6; with and without allergic rhinitis on examination; with negative and positive antibodies; with and without IVIG treatment; and with and without ICU admission (p > 0.05) (Table 5).

The MGFA score in the group with generalized MG was significantly higher than in the group with ocular MG (p < 0.05) and the MGFA score in the thymectomy group was significantly (p < 0.05) higher than in the nonthymectomy group (Table 5).

The MG-QOL15-T scores did not differ significantly between the groups with and without McGill–Melzack pain; with a normal and impaired MoCA degree; with inactive and minimally active physical activity; with and without normal olfactory function; with odor detection at concentrations ≤5 and >6; with and without allergic rhinitis on examination; with negative and positive antibodies; with and without thymectomy; with and without IVIG treatment; and with and without ICU admission (p > 0.05) (Table 6).

The MG-QOL15-T score in the group with generalized MG was significantly higher than in the group with ocular type MG (p < 0.05). The MG-QOL15-T score in the depression group was significantly higher than in the nondepression group (p < 0.05). Similarly, the MG-QOL15-T score in the anxiety group was significantly higher than in the nonanxiety group (p < 0.05). In the group with poor PSQI scores, the MG-QOL15-T score was significantly higher than in the group with normal PSQI scores (p < 0.05) (Table 6). The MG-QOL15-T score in the headache group was significantly lower than in the group without headaches (p < 0.05) (Table 6).

There was no significant difference in the MG disease duration between the groups with and without headache; with and without McGill–Melzack pain; with a normal and impaired MoCA degree; with inactive and minimally active physical activity; with and without depression; with the anxiety and the nonanxiety; with normal and poor PSQI scores; with and without normal olfactory function; with odor detection at concentrations ≤5 and >6; with and without allergic rhinitis on examination; with ocular and generalized MG; with negative and positive antibodies; with and without thymectomy; with and without IVIG treatment; and with and without ICU admission (p > 0.05) (Table 7).

All the participants scored 6 on the Katz ADL and were fully independent. Therefore, the scale was not included in the statistics.

## 4. Discussion

In MG patients, motor symptoms are frequently evaluated clinically, and treatment regulations are directed towards the motor symptoms. Nonmotor symptoms belonging to different systems are often not questioned or overlooked [2,5]. It was stated that many different nonmotor symptoms, in addition to fatigue and skeletal muscle weakness, may also be observed during the course of the disease. These symptoms negatively affect both the course of the disease and the patient’s daily life activities [15].

A few studies in the literature have evaluated nonmotor symptoms, especially pain and autonomic findings [12,13]. Nonmotor findings seen in MG range from myocarditis and red cell aplasia to pain and loss of smell [12–14,16,35,36]. In one study where pain complaints were investigated, 68 MG patients were evaluated, and 50% had pain complaints [42]. In a study evaluating headache in MG patients, tension-type headache was found in 38.6% of 184 patients and migraine was found in 4.9%. It was stated that headache started or worsened after the MG onset in 13.6% [43]. In recent years, different cardiac disorders such as acute heart failure, like acute coronary syndrome, myocarditis, and Takotsubo cardiomyopathy, have been described in MG patients, especially in the elderly and those with multiple drug usage, and the evaluation of autonomic findings among nonmotor symptoms has become important in this process [44]. In the current study, the headache rate in the patient group was significantly higher (51.4%) than in the control group; however, the McGill–Melzack pain rate did not differ significantly between the control and patient groups.

The findings of the current study underscore the significant prevalence and diverse spectrum of nonmotor symptoms in patients with MG, reinforcing the notion that MG is not merely a disorder of neuromuscular transmission but a systemic autoimmune condition with multifaceted manifestations. Consistent with previous studies [12,25,26], the data herein revealed that nonmotor symptoms, including but not limited to autonomic dysfunctions, sensory impairments, and systemic autoimmune phenomena, are common in MG patients, affecting a considerable proportion of the cohort. These nonmotor features, which may be encountered in patients with MG, can also be seen as systemic manifestations like sleep disorders, cognitive dysfunction, and psychosocial and mental health issues [12]. Herein, the BDI, BAI, and PSQI scores in the patient group were significantly higher than in the control group, which means that the depression, anxiety, and poor sleep quality rates in the patient group were significantly higher than in the control group. The MoCA score in the patient group was significantly lower than in the control group, which means that the impaired cognitive function rate in the patient group was significantly higher than in the control group.

The observation of widespread nonmotor symptoms aligns with the findings of Suzuki et al. [13], Suzuki [14], and Uzawa et al. [16], which highlighted the presence of symptoms beyond muscle weakness, such as alopecia areata and taste disorders, particularly prevalent among patients with thymoma-associated MG. In studies conducted in Japan, it was stated that approximately 25% of thymoma-related MG patients had at least one nonmotor symptom, and alopecia areata and taste disorders ranked first [13,14]. This association points toward a wide immune dysregulation in MG, potentially implicating additional autoimmune mechanisms beyond the well-characterized antibodies against the acetylcholine receptor. In the current study, in the patient group, 19 had a history of COVID-19 infection, 10 had a history of rhinitis, and one had a history of nasal trauma. In the control group, 17 had a history of COVID-19 infection, nine had a history of rhinitis, and two had a history of nasal trauma. Therefore, unlike the literature, there was no statistically significant difference between the patient and control groups in terms of taste function (bitter, sour, sweet, and salty). However, a marginal decrease in bitter taste function (45.7%) was observed in the patient group compared to the control group, which was not statistically significant.

The present research contributes further to this narrative by establishing a correlation between the severity of MG and the incidence of nonmotor symptoms, particularly olfactory dysfunction. This correlation supports the proposition that the degree of immune dysregulation might correlate with the clinical severity of MG, an insight that resonates with findings from studies indicating reduced olfactory performance in MG patients compared to controls [35,36]. In a study evaluating olfactory functions, the olfactory performance of MG patients was lower than that of polymyositis patients and the control group, and the loss of smell was correlated with the severity of MG symptoms [36]. Acetylcholine is the major neurotransmitter in the efferent transmission of hair cells in the cochlea. Relatedly, cochlear functions are also affected in MG patients [45]. Such findings provide a nuanced understanding of MG, where sensory and autonomic functions are considered integral to disease pathology and patient care. In the present study, unlike the literature, there was no statistically significant difference in olfactory function between the patient and control groups. However, olfactory function with cinnamon in the patient group was significantly lower (25.7%) than in the control group.

The pathogenic mechanisms of nonmotor symptoms in MG remain complex and multifactorial. The data herein lend support to the hypothesis of antibody-mediated damage and CD8+ T cell-mediated cytotoxicity as contributory mechanisms [13–15]. These findings are in line with the broader literature on autoimmune diseases, where a confluence of humoral and cellular immune responses underlies various clinical manifestations [17,25,27,29]. Such mechanisms in MG not only provide a potential explanation for the wide arrangement of nonmotor symptoms observed, but also open routes for precise therapeutic interventions, including plasmapheresis (PE) and IVIG, which have shown efficacy in neuromyotonia and limbic encephalitis associated with MG [13,16,25,26]. Hence, two different pathogenesis related to nonmotor findings were stated. These were antibody-related damage and CD8+ T cell ototoxicity. Neuromyotonia, limbic encephalitis, myocarditis, and taste disorders are responsive to PE and IVIG treatment. These clinical presentations are also associated with MG activation. Therefore, it is thought that there is an autoantibody-related immune response here. Alopecia areata, red cell aplasia, and immune deficit-related diseases are independent of the MG clinic and the response of these diseases to immune therapy is limited [13,14,16]. In the present study, no relationship was observed between MGC, MGFA, and MG-QOL15-T scores and MG disease duration in terms of receiving IVIG therapy and admission to the ICU. However, the MGFA score in the thymectomy group was significantly higher than in the nonthymectomy group.

This study’s findings have important clinical implications. They highlight the necessity for clinicians to adopt a comprehensive approach to MG management, one that encompasses not only the treatment of motor symptoms but also the identification and administration of nonmotor symptoms. Such an approach is crucial for improving the quality of life in MG patients, given the prominent impact of nonmotor symptoms on daily functioning and well-being.

In conclusion, the prevalent and several nonmotor symptoms identified in the current MG population emphasize the systemic nature of MG and its effect beyond neuromuscular junction impairment. These findings advocate for a paradigm shift in MG management, highlighting the need for inclusive symptom assessment and a multidisciplinary treatment strategy. Further research is needed to explain the pathogenic mechanisms underlying these nonmotor manifestations, which will be pivotal in developing more targeted and effective therapeutic strategies.

There were a few limitations that should be considered. First, the small sample size necessitates larger, controlled trials to confirm the generalizability of these findings. The sample size was relatively small, potentially limiting the generalizability of the findings. Future studies with larger sample sizes are needed to further validate the results. Additionally, individual factors might have influenced outcomes, warranting further exploration.

This comprehensive evaluation of nonmotor symptoms in MG patients has illuminated the substantial prevalence and wide spectrum of these manifestations, extending beyond the primary neuromuscular symptoms to include autonomic, sensory, and systemic autoimmune phenomena. These findings challenge the traditional view of MG as a disorder confined to muscle weakness and fatigue, highlighting its efficacy on a broader array of physiological systems.

The significant correlation between the severity of MG and the presence of nonmotor symptoms, particularly sensory impairments like olfactory dysfunction, underscores the systemic nature of MG and suggests a complex interplay between immune dysregulation and clinical manifestations. This strengthens the need for a holistic approach to MG management, one that recognizes and addresses the full spectrum of patient symptoms to improve overall quality of life.

This study also points to the potential basic mechanisms of nonmotor symptoms in MG, including antibody-mediated damage and CD8+ T cell cytotoxicity, offering avenues for targeted therapeutic interventions. The efficacy of treatments such as PE and IVIG in managing neuromyotonia and limbic encephalitis associated with MG emphasizes the possibility of extending these therapies to a large range of nonmotor symptoms.

However, the pathogenesis of nonmotor symptoms in MG remains incompletely understood, and our findings underline the need for further research in this area. Future studies should aim to elucidate the molecular and cellular mechanisms underlying these symptoms, explore the effectiveness of novel therapeutic options, and assess the impact of extensive symptom administration on patient outcomes.

In conclusion, this study emphasizes the importance of recognizing and managing nonmotor symptoms in MG patients, advocating for a multidisciplinary policy to care those addresses both motor and nonmotor manifestations. By expanding our understanding of MG’s systemic influence and exploring new treatment modalities, we can improve the lives of those living with this complicated autoimmune disorder.

## Figures and Tables

**Table 1:** Clinical characteristics of the patient group.

		Min–max			Median	Mean ± SD/n-%		
MG type	Ocular					15		42.9%
	Generalized					20		57.1%
MGC score		0.0	–	17.0	2.0	4.5	±	4.8
MGFA score		0.0	–	7.0	1.0	1.9	±	2.0
MG-QOL15-T score		0.0	–	45.0	7.0	11.1	±	11.9
MG disease duration (year)		1.0	–	25.0	5.0	6.4	±	6.2
Antibody	Negative					6		17.1%
	Positive					29		82.9%
Thymectomy	(−)					11		31.4%
	(+)					24		68.6%
IVIG	(+)					13		37.1%
	(−)					22		62.9%
Admission to the ICU	(+)					3		8.6%
	(−)					32		91.4%

**Table 2:** Comparison of the demographic characteristics and nonmotor symptoms of MG in the patient and control groups.

		Control group (n = 35)				Patient group (n = 35)				p	
		Mean ± SD/n-%			Median	Mean ± SD/n-%			Median		
Age		46.9	±	12.2	51.0	52.9	±	13.6	54.0	0.064	m
Sex	Female	23		65.7%		17		48.6%		0.147	X^2^
	Male	12		34.3%		18		51.4%			
Comorbidity	(+)	2		5.7%		8		22.9%		***0.040***	X^2^
	(−)	33		94.3%		27		77.1%			
MoCA Score		28.2	±	2.3	29.0	25.0	±	5.2	27.0	***0.004***	m
MoCA Degree	Normal	35		100.0%		28		80.0%		***0.005***	X^2^
	Impaired	0		0.0%		7		20.0%			
BDI score		7.2	±	5.0	7.0	11.6	±	8.8	10.0	***0.030***	m
Presence of depression	(−)	28		80.0%		14		40.0%		***0.001***	X^2^
	(+)	7		20.0%		21		60.0%			
BAI score		3.8	±	3.0	3.0	9.1	±	9.9	7.0	***0.010***	m
Presence of anxiety	(−)	30		85.7%		19		54.3%		***0.004***	X^2^
	(+)	5		14.3%		16		45.7%			
PSQI Score		2.4	±	1.5	2.0	5.0	±	3.2	4.0	***0.000***	m
Sleep quality	Normal	33		94.3%		19		54.3%		***0.000***	X^2^
	Poor	2		5.7%		16		45.7%			
Olfactory function	Normal	13		37.1%		14		40.0%		0.806	X^2^
	Impaired	22		62.9%		21		60.0%			
Odor concentration		6.4	±	1.4	6.0	5.8	±	1.6	6.0	0.211	m

m Mann–Whitney U test,

X2 chi-squared test (Fischer’s test).

**Table 3:** Comparison of the nonmotor symptoms MG in the control and patient groups.

		Control group (n = 35)				Patient group (n = 35)				p	
		Mean ± SD/n-%			Median	Mean ± SD/n-%			Median		
Headache	(−)	32		91.4%		17		48.6%		***0.000***	X^2^
	(+)	3		8.6%		18		51.4%			
Physical activity	Inactive	16		45.7%		15		42.9%		0.810	X^2^
	Minimal Active	19		54.3%		20		57.1%			
McGill–Melzack Pain	(+)	17		48.6%		17		48.6%		1.000	X^2^
	(−)	18		51.4%		18		51.4%			
***Olfactory function***											
Baby powder		0		0.0%		1		2.9%		1.000	X^2^
Coffee		25		71.4%		21		60.0%		0.314	X^2^
Vicks		32		91.4%		29		82.9%		0.284	X^2^
Cinnamon		23		65.7%		9		25.7%		***0.001***	X^2^
Cocoa		14		40.0%		17		48.6%		0.470	X^2^
Soap		29		82.9%		23		65.7%		0.101	X^2^
Naphthalene		20		57.1%		20		57.1%		1.000	X^2^
Peanut Butter		14		40.0%		18		51.4%		0.337	X^2^
***Taste function***											
Bitter		24		68.6%		16		45.7%		0.053	X^2^
Sour		21		60.0%		23		65.7%		0.621	X^2^
Sweet		27		77.1%		27		77.1%		1.000	X^2^
Salty		27		77.1%		27		77.1%		1.000	X^2^
Allergic rhinitis on examination	(+)	3		8.6%		5		14.3%		0.452	X^2^
	(−)	32		91.4%		30		85.7%			

m Mann–Whitney U test,

X2 chi-squared test (Fischer’s test).

**Table 4:** Relationship between the composite scores and the nonmotor symptoms/clinical features of MG.

		MGC score							p	
		Min–max			Median	Mean ± SD				
Headache	(−) (n: 49)	0.00	–	17.00	5.00	5.88	±	5.36	0.096	m
	(+) (n: 21)	0.00	–	15.00	2.00	3.28	±	3.83		
McGill–Melzack pain	(−) (n: 36)	0.00	–	17.00	3.00	4.72	±	4.79	0.527	m
	(+) (n: 34)	0.00	–	15.00	2.00	4.35	±	4.86		
MoCA degree	Normal (n: 63)	0.00	–	17.00	2.00	4.11	±	4.80	0.134	m
	Impaired (n: 7)	1.00	–	15.00	5.00	6.29	±	4.46		
Physical activity	Inactive (n: 31)	0.00	–	17.00	2.00	4.73	±	5.43	0.920	m
	Minimal active (n: 39)	0.00	–	15.00	3.50	4.40	±	4.32		
Presence of depression	(−) (n: 42)	0.00	–	15.00	1.50	2.79	±	4.14	***0.023***	m
	(+) (n: 28)	0.00	–	17.00	5.00	5.71	±	4.87		
Presence of anxiety	(−) (n: 49)	0.00	–	15.00	2.00	3.58	±	4.03	0.256	m
	(+) (n: 21)	0.00	–	17.00	5.50	5.69	±	5.40		
PSQI score	Normal (n: 52)	0.00	–	17.00	4.00	4.89	±	4.99	0.738	m
	Poor (n: 18)	0.00	–	15.00	2.00	4.13	±	4.59		
Olfactory function	Normal (n: 27)	0.00	–	17.00	4.00	5.43	±	5.26	0.300	m
	Impaired (n: 43)	0.00	–	15.00	2.00	3.95	±	4.42		
Odor concentration	≤5 (n: 20)	0.00	–	17.00	5.00	6.15	±	5.67	0.248	m
	>6 (n: 50)	0.00	–	15.00	2.00	3.59	±	3.96		
Allergic rhinitis on examination	(−) (n: 62)	0.00	–	17.00	4.00	5.17	±	4.82	***0.010***	m
	(+) (n: 8)	0.00	–	4.00	0.00	0.80	±	1.79		
MG type	Ocular (n: 15)	0.00	–	15.00	2.00	3.27	±	3.94	0.253	m
	Generalized (n: 20)	0.00	–	17.00	5.00	5.50	±	5.18		
Antibody	Negative (n: 6)	0.00	–	11.00	3.50	4.33	±	4.18	0.947	m
	Positive (n: 29)	0.00	–	17.00	2.00	4.59	±	4.93		
Thymectomy	(−) (n: 24)	0.00	–	17.00	2.00	4.58	±	4.94	0.957	m
	(+) (n: 11)	0.00	–	15.00	4.00	4.45	±	4.55		
IVIG	(−) (n: 22)	0.00	–	17.00	2.00	4.41	±	4.85	0.796	m
	(+) (n: 13)	0.00	–	15.00	4.00	4.77	±	4.78		
Admission to the ICU	(−) (n: 32)	0.00	–	17.00	2.00	4.34	±	4.56	0.572	m
	(+) (n: 3)	1.00	–	15.00	4.00	6.67	±	7.37		

m Mann–Whitney U test.

**Table 5:** Relationship between the MGFA Scores and the nonmotor symptoms/clinical features of MG.

		MGFA score							p	
		Min–max			Median	Mean ± SD				
Headache	(−) (n: 49)	0.00	–	7.00	2.00	2.06	±	2.25	0.892	m
	(+) (n: 21)	0.00	–	6.00	1.00	1.72	±	1.67		
McGill–Melzack pain	(−) (n: 36)	0.00	–	7.00	1.00	1.72	±	1.87	0.710	m
	(+) (n: 34)	0.00	–	6.00	2.00	2.06	±	2.08		
MoCA degree	Normal (n: 63)	0.00	–	7.00	1.00	1.68	±	1.81	0.301	m
	Impaired (n: 7)	0.00	–	6.00	2.00	2.71	±	2.43		
Physical activity	Inactive (n: 31)	0.00	–	7.00	1.00	1.87	±	2.10	0.838	m
	Minimal active (n: 39)	0.00	–	6.00	1.50	1.90	±	1.89		
Presence of depression	(−) (n: 42)	0.00	–	6.00	1.00	1.64	±	1.69	0.705	m
	(+) (n: 28)	0.00	–	7.00	2.00	2.05	±	2.13		
Presence of anxiety	(−) (n: 49)	0.00	–	6.00	1.00	1.32	±	1.60	0.053	m
	(+) (n: 21)	0.00	–	7.00	2.00	2.56	±	2.16		
PSQI score	Normal (n: 52)	0.00	–	7.00	1.00	1.63	±	2.14	0.150	m
	Poor (n: 18)	0.00	–	6.00	2.00	2.19	±	1.72		
Olfactory function	Normal (n: 27)	0.00	–	7.00	1.00	2.14	±	2.14	0.558	m
	Impaired (n: 43)	0.00	–	6.00	2.00	1.71	±	1.85		
Odor concentration	≤5 (n: 20)	0.00	–	7.00	2.00	2.23	±	2.13	0.382	m
	>6 (n: 50)	0.00	–	6.00	1.00	1.68	±	1.86		
Allergic rhinitis on examination	(−) (n: 62)	0.00	–	7.00	2.00	2.07	±	2.00	0.135	m
	(+) (n: 8)	0.00	–	3.00	0.00	0.80	±	1.30		
MG type	Ocular (n: 15)	0.00	–	3.00	0.00	0.80	±	1.01	***0.003***	m
	Generalized (n: 20)	0.00	–	7.00	2.00	2.70	±	2.11		
Antibody	Negative (n: 6)	0.00	–	2.00	0.50	0.67	±	0.82	0.070	m
	Positive (n: 29)	0.00	–	7.00	2.00	2.14	±	2.03		
Thymectomy	(−) (n: 24)	0.00	–	7.00	1.00	1.42	±	1.74	***0.028***	m
	(+) (n: 11)	1.00	–	6.00	2.00	2.91	±	2.07		
IVIG	(−) (n: 22)	0.00	–	7.00	1.00	1.73	±	1.98	0.421	m
	(+) (n: 13)	0.00	–	6.00	2.00	2.15	±	1.95		
Admission to the ICU	(−) (n: 32)	0.00	–	7.00	1.00	1.78	±	1.90	0.350	m
	(+) (n: 3)	1.00	–	6.00	2.00	3.00	±	2.65		

m Mann–Whitney U test.

**Table 6:** Relationship between the MG-QOL15-T scores and the nonmotor symptoms/clinical features of MG.

		MG-QOL15-T score							p	
		Min–max			Median	Mean ± SD				
Headache	(−) (n: 49)	0.0	–	45.0	9.0	15.5	±	13.4	***0.042***	m
	(+) (n: 21)	0.0	–	39.0	5.5	7.0	±	8.7		
McGill–Melzack pain	(−) (n: 36)	0.0	–	45.0	5.5	10.3	±	13.5	0.254	m
	(+) (n: 34)	0.0	–	34.0	8.0	12.0	±	10.2		
MoCA degree	Normal (n: 63)	0.0	–	45.0	6.5	10.6	±	12.0	0.577	m
	Impaired (n: 7)	2.0	–	31.0	8.0	13.0	±	12.1		
Physical activity	Inactive (n: 31)	0.0	–	45.0	7.0	10.8	±	12.5	0.841	m
	Minimal active (n: 39)	0.0	–	39.0	8.0	11.4	±	11.7		
Presence of depression	(−) (n: 42)	0.0	–	13.0	4.0	4.4	±	4.0	***0.003***	m
	(+) (n: 28)	0.0	–	45.0	10.0	15.6	±	13.3		
Presence of anxiety	(−) (n: 49)	0.0	–	13.0	6.0	5.6	±	4.1	***0.024***	m
	(+) (n: 21)	0.0	–	45.0	12.5	17.6	±	14.7		
PSQI score	Normal (n: 52)	0.0	–	45.0	5.0	8.6	±	12.3	***0.037***	m
	Poor (n: 18)	0.0	–	34.0	10.0	14.1	±	10.9		
Olfactory function	Normal (n: 27)	0.0	–	45.0	7.5	13.0	±	14.5	0.723	m
	Impaired (n: 43)	0.0	–	34.0	7.0	9.9	±	9.9		
Odor concentration	≤5 (n: 20)	0.0	–	45.0	7.0	12.3	±	13.5	0.771	m
	>6 (n: 50)	0.0	–	39.0	7.5	10.4	±	11.1		
Allergic rhinitis on examination	(−) (n: 62)	0.0	–	45.0	8.0	12.0	±	12.5	0.299	m
	(+) (n: 8)	0.0	–	13.0	5.0	5.6	±	4.9		
MG type	Ocular (n: 15)	0.0	–	13.0	4.0	4.3	±	4.2	***0.001***	m
	Generalized (n: 20)	3.0	–	45.0	9.5	16.2	±	13.2		
Antibody	Negative (n: 6)	0.0	–	34.0	7.0	10.5	±	12.0	0.983	m
	Positive (n: 29)	0.0	–	45.0	7.0	11.2	±	12.0		
Thymectomy	(−) (n: 24)	0.0	–	45.0	6.5	11.0	±	12.4	0.735	m
	(+) (n: 11)	2.0	–	34.0	8.0	11.4	±	11.2		
IVIG	(−) (n: 22)	0.0	–	45.0	6.5	10.4	±	12.6	0.472	m
	(+) (n: 13)	2.0	–	31.0	8.0	12.3	±	10.8		
Admission to the ICU	(−) (n: 32)	0.0	–	45.0	7.0	10.9	±	11.8	0.790	m
	(+) (n: 3)	2.0	–	31.0	8.0	13.7	±	15.3		

m Mann–Whitney U test.

**Table 7:** Relationship between the disease duration and nonmotor symptoms/clinical features of MG.

		MG disease duration							p	
		Min–max			Median	Mean ± SD				
Headache	(−) (n: 49)	1.00	–	25.00	5.00	6.65	±	7.09	0.665	m
	(+) (n: 21)	1.00	–	19.00	4.00	6.17	±	5.35		
McGill–Melzack pain	(−) (n: 36)	1.00	–	19.00	5.00	7.00	±	6.11	0.359	m
	(+) (n: 34)	1.00	–	25.00	3.00	5.76	±	6.35		
MoCA degree	Normal (n: 63)	1.00	–	19.00	4.00	5.82	±	5.17	0.532	m
	Impaired (n: 7)	1.00	–	25.00	5.00	8.71	±	9.34		
Physical activity	Inactive (n: 31)	1.00	–	16.00	6.00	7.07	±	5.16	0.297	m
	Minimal active (n: 39)	1.00	–	25.00	3.00	5.90	±	6.91		
Presence of depression	(−) (n: 42)	1.00	–	14.00	3.00	4.00	±	3.53	0.118	m
	(+) (n: 28)	1.00	–	25.00	5.00	8.00	±	7.06		
Presence of anxiety	(−) (n: 49)	1.00	–	16.00	3.00	5.11	±	4.34	0.493	m
	(+) (n: 21)	1.00	–	25.00	5.00	7.94	±	7.67		
PSQI score	Normal (n: 52)	1.00	–	25.00	3.00	6.05	±	6.47	0.815	m
	Poor (n: 18)	1.00	–	19.00	6.00	6.81	±	5.97		
Olfactory function	Normal (n: 27)	1.00	–	19.00	6.50	7.93	±	7.05	0.563	m
	Impaired (n: 43)	1.00	–	25.00	3.00	5.38	±	5.44		
Odor concentration	≤5 (n: 20)	1.00	–	25.00	3.00	6.15	±	6.85	0.629	m
	>6 (n: 50)	1.00	–	19.00	5.00	6.55	±	5.89		
Allergic rhinitis on examination	(−) (n: 62)	1.00	–	25.00	4.00	6.70	±	6.52	0.757	m
	(+) (n: 8)	1.00	–	9.00	5.00	4.60	±	3.21		
MG type	Ocular (n: 15)	1.00	–	14.00	5.00	5.73	±	4.10	0.711	m
	Generalized (n: 20)	1.00	–	25.00	3.00	6.90	±	7.42		
Antibody	Negative (n: 6)	1.00	–	25.00	4.00	8.67	±	9.75	0.791	m
	Positive (n: 29)	1.00	–	19.00	5.00	5.93	±	5.28		
Thymectomy	(−) (n: 24)	1.00	–	25.00	5.00	7.00	±	6.72	0.484	m
	(+) (n: 11)	1.00	–	14.00	3.00	5.09	±	4.74		
IVIG	(−) (n: 22)	1.00	–	25.00	3.00	5.95	±	6.69	0.179	m
	(+) (n: 13)	1.00	–	19.00	5.00	7.15	±	5.34		
Admission to the ICU	(−) (n: 32)	1.00	–	25.00	4.00	6.38	±	6.23	0.953	m
	(+) (n: 3)	1.00	–	14.00	5.00	6.67	±	6.66		

m Mann–Whitney U test.
